# Target Discovery in Head-and-Neck Squamous Cell Carcinoma: Genome-Wide CRISPR Screens Illuminate Therapeutic Resistance and Actionable Dependencies

**DOI:** 10.3390/biomedicines13123012

**Published:** 2025-12-08

**Authors:** Vui King Vincent-Chong

**Affiliations:** Department of Oral Oncology, Roswell Park Comprehensive Cancer Center, Buffalo, NY 14263, USA; vincentvuiking.chong@roswellpark.org

**Keywords:** oral squamous cell carcinoma, OSCC, head-and-neck squamous cell carcinoma, HNSCC, genome-wide CRISPR cas9 knockout screen, cisplatin, radiation, resistance, preclinical model, vulnerabilities

## Abstract

Head-and-neck squamous cell carcinoma (HNSCC) remains a lethal malignancy with stagnant survival despite advances in surgery, radiotherapy, and systemic therapy. Beyond cetuximab and PD-1 inhibitors, there are only a few targeted options, which benefit only a minority of patients, underscoring the need for new biomarkers and druggable dependencies. Genome-wide clustered regularly interspaced short palindromic repeats (CRISPR) Cas9 screening now enables systematic, high-specificity investigation of gene function to reveal determinants of tumor proliferation, survival, and therapy response. Compared with RNA interference, CRISPR provides cleaner on-target knockout and more interpretable phenotypes, allowing efficient discovery of essential genes and synthetic-lethal interactions. Although the Cancer Dependency Map profiled 89 OSCC/HNSCC lines to nominate baseline dependencies, drug-perturbed states critical for understanding platinum resistance remain underexplored. Only a handful of HNSCC studies have applied genome-wide CRISPR cas9 screening: two mapped core essential genes; two mapped cisplatin resistance and radiation resistance; and others uncovered synthetic-lethal targets, including vulnerabilities to mTOR inhibition, EGFR inhibition, glutamine metabolism inhibition, and host determinants of oncolytic HSV-1 efficacy. This review synthesizes these findings, highlights methodological considerations (library design, coverage, and treatment duration), and integrates complementary functional data to prioritize targets for rational combinations. This review also provides information on the TCGA database and in vivo CRISPR screening that can accelerate precision therapeutics for patients with HNSCC.

## 1. Introduction

Head-and-neck squamous cell carcinomas (HNSCCs) are aggressive malignancies that afflict more than 800,000 people worldwide [1,2]. In the United States, HNSCCs were listed among the top 10 leading cancers diagnosed in men in 2025, and the incidence has continued to increase at a rate of approximately 1% annually [2]. In contrast, the survival rate has hardly changed for these patients over the past two decades despite advances in surgical techniques, chemotherapies, and radiotherapy technology, and HNSCC patients for whom standard-of-care treatments (such as platinum-based chemotherapy) failed currently have a median survival rate of less than one year, highlighting the critical need to develop better therapeutic strategies to improve patients’ clinical outcomes [2,3]. Recent progress along the drug discovery and development pipeline for HNSCC has been incremental. Since 2006, the EGFR-targeting cetuximab has remained the only approved molecular-targeted drug for HNSCC [4]. In recent years, two anti PD-1 immunotherapy drugs (pembrolizumab and nivolumab) have been approved for the treatment of recurrent/ metastatic HNSCC patients for whom concurrent chemoradiotherapy (CRT) regimens fail [5,6]. However, despite these advancements, only a minority of patients respond to these drugs, underscoring the unmet need to develop novel prognosticators and therapeutic targets.

Clustered Regularly Interspaced Short Palindromic Repeats (CRISPR) is an adaptive immune system that is used by bacteria and archaea for defense against viruses and other forms of foreign DNA [7]. Due to its ability to recognize and destroy foreign DNA, this system has been repurposed as a versatile genome-engineering tool for biomedical science research [8]. The advantages of CRISPR include its simplicity and fast target site design, accurate and specific gene targeting, efficiency as a genome-editing tool, and ability to modify several different genes simultaneously. Briefly, the CRISPR Cas9 system comprises an RNA-guided nuclease (either Cas9 or a similar enzyme) and a single-guide RNA (sgRNA), which target and cleave specific DNA sequences to introduce site-specific DNA breaks and mutations [9]. The dCas9, known as the nuclease-dead variant, can fuse with transcriptional activators or repressors, leading to modulated gene expression by either increasing (CRISPRa) or decreasing (CRISPRi) gene expression without cutting the DNA [10].

Following recent advances, genome-wide CRISPR Cas9 screening now enables systematic assessment of how gene knockouts (KOs) affect cell proliferation and gene–drug interactions [11]. This approach involves pooled libraries spanning thousands of loci, allowing for genome-wide screening. This high-throughput approach allows us to investigate thousands of genes simultaneously in a population of cells through perturbation, as the pooled library of guide RNAs enables systematic discovery of genes regulating proliferation, drug response, and survival. Earlier efforts involved the use of RNA interference (RNAi), but its lower target specificity and variable knockdown efficiency limited interpretability [12]. Consequently, an increasing number of studies now employ CRISPR Cas9 to identify essential genes, uncover druggable vulnerabilities, and delineate mechanisms of action in depth. In summary, the development of guide RNA libraries targeting thousands of genes facilitates the production of cluster of cells that contain a single KO for genetic screening. This allows the efficient identification of genes involved in tumor proliferation, survival, and therapeutic resistance, providing us with the vulnerabilities and therapeutic targets that are potentially associated with poor prognosis [11].

**Figure 1:**
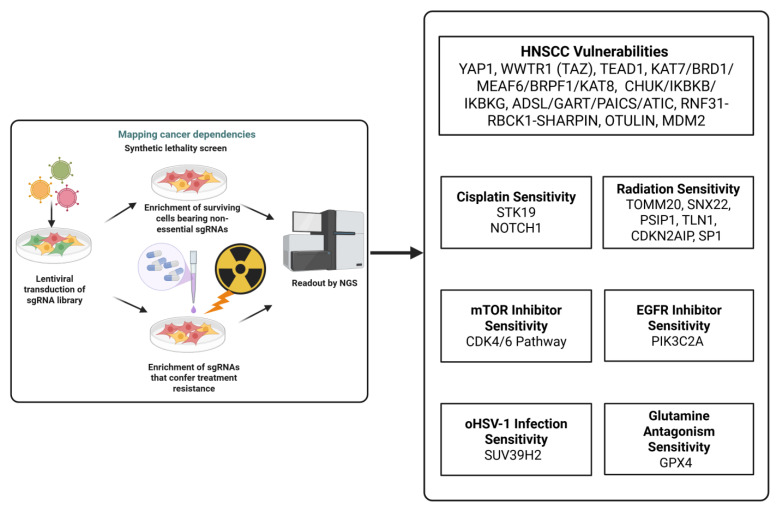
This figure illustrates the integration between essential gene discovery and therapy resistance and highlights the representative dependency or resistance genes in cisplatin, radiation, and targeted therapy for HNSCC. Created in Biorender Vincent-Chong, V.K. (2025). https://BioRender.com/6hnc0sl.

To date, fewer than 20 studies have employed genome-wide CRISPR Cas9 screens in HNSCC in vitro. Two studies mapped genes essential for HNSCC cell proliferation and survival [13,14]; two specifically interrogated cisplatin resistance [15,16]; one study investigated radiation resistance [17]; and three focused on synthetic-lethal targets, covering vulnerabilities to mTOR [18] or EGFR [19] or glutamine metabolism [20] inhibitors/antagonists and a host determinant of oncolytic HSV-1 (oHSV-1) [21] efficacy, respectively. In 2019, the Cancer Dependency Map (DepMap) profiled 89 OSCC/HNSCC lines with CRISPR Cas9 to nominate candidate therapeutic targets [22]. However, because DepMap screens assess baseline genetic dependency rather than drug-perturbed states, they did not reveal novel cisplatin-resistance genes, as cisplatin’s effects arise under treatment conditions that were not modeled in those assays. To address this knowledge gap, this review outlines (i) essential genes in OSCC/HNSCC identified via genome-wide CRISPR KO, (ii) genes implicated in cisplatin/radiation resistance, and (iii) other types of therapeutic resistance found through genome-wide CRISPR screens and complementary functional studies, highlighting translational opportunities for target prioritization and combination therapy design. The details of the sgRNA library and the approaches taken in each study are illustrated in Table 1. Figure 1 provides an illustration of how lentiviral sgRNA library transduction and next-generation sequencing (NGS) readouts are used to identify cancer dependencies and both baseline essential genes and treatment-specific vulnerabilities.

## 2. Research Strategy

PubMed was searched from 2019 to 2025 using various combinations of the following keywords: “oral squamous cell carcinoma (OSCC)” and “head and neck squamous cell carcinoma (HNSCC)”, “genome wide CRISPR Cas9 screening”, “chemotherapy”, “cisplatin”, “radiation therapy”, “treatment resistance”, and “therapeutic resistance”. Studies conducted prior to 2021 were also included as these studies provide us with the foundational insights for CRISPR screens that helped us increase the scale of functional genomic research on HNSCC. Original experimental studies from published in English were considered and included.

## 3. Results

HNSCC/OSCC remains one of the major public health malignancies worldwide due to the high propensity for lymph node metastasis (locoregional advanced disease) and the poor prognosis for patients [23]. Therefore, novel therapeutic strategies that target these malignancies’ dependency genes enable suppression of their growth and promotion of cell death to improve patients’ prognoses. Genome-wide CRISPR Cas9 KO (dropout) screening can systematically discover genes essential for tumor cell proliferation and survival and allow us to target these genes to improve clinical outcomes. This platform also lets us identify genes that drop out under a given treatment. Critically, these genes represent sensitizers or synthetic-lethal partners, because tumor cells rely on them to survive therapeutic interactions [11,24].

### 3.1. Essential Genes in OSCC/HNSCC Identified via Genome-Wide CRISPR Knockout

Through the establishment of a DepMap genome-scale CRISPR knockout on a panel of 89 HNSCC cell lines, this project allowed us to identify a panel of essential genes from bona fide cell lines that are studied in HNSCC research [22]. However, few representative cell lines from the Asian population have been established. Critically, one of the risky habits attributed to HNSCC is chewing betel quid, a rare practice in Western countries. With this in mind, Chai et al. [13] performed genome-wide CRISPR knockout screening on a panel of 14 HNSCC cell lines derived from Malaysian HNSCC patients and 7 OSCC cell lines sourced from commercial cell line repositories to identify the vulnerability gene essential to HNSCC cell survival so that novel candidate-gene-targeted therapies could be developed to improve clinical outcomes. Their study revealed 918 fitness genes, including YAP1 and WWTR1 (TAZ)—two paralogous transcription co-activators in the Hippo pathway. Instead of universal essentiality, the study discovered differential and compensable dependencies of a subset of OSCC cell lines that depended on YAP1 or WWTR1, while others were not dependent. To determine vulnerability, they compared knockout models of YAP1A and WWTR1 with the wild type in vitro to determine their cell proliferation and survival capacity, and they identified that knocking out both genes reduces cell proliferation and enhances cell apoptosis only in their respective dependent cell lines. These observations highlight the significance of considering redundancy and dependency heterogeneity when analyzing CRISPR data due to the possibility of acquiring a panel of genes featuring context-dependent vulnerabilities instead of generally essential genes. The limitation of this study is that the authors did not validate this notion using an in vivo preclinical model, which could have enabled further therapeutic target development. The DepMap project has profiled 89 HNSCC cell lines derived from diverse subsites, such as buccal mucosa, the tongue, the floor of the mouth, the tonsils, the larynx, the pharynx, the oropharynx, the hypopharynx, gingiva, and salivary glands, and reported the top preferential essential genes (depmap.org/portal) [22]. However, none of these cell lines originate from the nasopharynx. To address this gap, Wang et al. [14] performed a genome-wide CRISPR screen of nasopharyngeal carcinoma (NPC) to identify vulnerability genes and elucidate mechanisms that promote NPC growth and survival in vitro. They identified 711 genes whose loss depleted NPC cells but not normal nasopharyngeal cells. These vulnerabilities included members of the MYST histone acetyltransferase family (KAT7, BRD1, MEAF6, BRPF1, and KAT8) as well as genes involved in NF-κB signaling (CHUK, IKBKB, and IKBKG), de novo purine synthesis (ADSL, GART, PAICS, and ATIC), linear ubiquitination (LUBAC (RNF31-RBCK1-SHARPIN) and OTULIN), and the TP53 pathway (MDM2). Targeted knockout of representative genes such as KAT7, BRD1, MEAF6, and KAT8 (acetyltransferases); CHUK, IKBKB, and IKBKG (NF-κB); ADSL and GART (purine synthesis); RNF31 and OTULIN (linear ubiquitination); and MDM2 (p53 control) reduced proliferation of NPC cells in vitro relative to normal nasopharyngeal controls, supporting their functional essentiality. Overall, these genome-wide CRISPR knockout screening studies have identified a panel of molecular dependencies that sustain tumor proliferation and survival. These genes are related to recurrently essential pathways that involve the Hippo signaling axis (YAP1/WWTR1), epigenetic regulators of the MYST acetyltransferase family (KAT7, BRD1, MEAF6, and KAT8), NF-κB signaling components (CHUK, IKBKB, and IKBKG), and enzymes involved in purine biosynthesis (ADSL, GART, PAICS, and ATIC). Therefore, targeting the genes that relate to these essential pathways either through functional or pharmacologic inhibition is warranted.

### 3.2. Genes Implicated in Cisplatin/Radiation Resistance from Genome-Wide CRISPR Screens

Despite advances in treatment, patients with HNSCC still have poor outcomes due to chemotherapy resistance [23]. Although cetuximab and immune checkpoint blockade are approved for use in treatment, their benefits are limited, underscoring the need for new strategies that improve response and survival. One powerful approach is genome-wide CRISPR dropout screening, which systematically tests how gene knockouts affect fitness under therapy. In this framework, genes whose loss causes selective dropout only in drug-treated cells (but not vehicles) are identified as sensitizers or synthetic-lethal partners, revealing pathways such as DNA repair, cell-cycle checkpoints, and anti-apoptotic signaling that tumors depend on to withstand the therapy in question. To date, two studies focusing on tongue squamous cell carcinoma (TSCC) have employed genome-wide CRISPR knockout (dropout) screening to identify genes that mediate or sensitize to cisplatin resistance. Li et al. [15] identified 17 kinases whose sgRNAs were significantly depleted after cisplatin exposure in two independent tongue SCC cell lines (TSCCA and CAL27) using a customized library (5171 sgRNAs targeting 508 kinases). On this basis, the authors identified STK19 as a cisplatin-sensitizing target in both TSCC cell lines and demonstrated that STK19 KO synergistically enhanced cisplatin cytotoxicity led to marked accumulation of DNA damage. STK19 expression also declined with an increasing cisplatin dose. Mechanistically, pharmacologic inhibition via T-12-037-01 (a potent STK19 inhibitor) enhanced cisplatin-induced DNA damage and was accompanied by MGMT depletion, indicating a synthetic-lethal interaction. This study reveals a comprehensive CRISPR validation pipeline in HNSCC research, combining both genetic and pharmacologic confirmation across multiple models. The cisplatin-and-T-12-037-01 combination induced synergistic tumor growth inhibition in vivo in a TSCCA cell line. In addition, Ludwig et al. [16] identified 207 genes whose depletion increased cisplatin sensitivity and found that NOTCH signaling was a top resistance pathway that is regulated by NOTCH1, SSPO, NCOR1, MARK2, and MYCBP based on a genome-wide CRISPR Cas9 screen of a TSCC cell line. They further demonstrated that genetically depleted NOTCH1 or pharmacological use of the γ-secretase inhibitor DAPT enhanced cisplatin sensitivity in vitro. Radiation resistance has been documented as a major determinant of poor outcomes in the context of HNSCC/NPC owing to pro-survival signaling and efficient DNA-damage repair. To elucidate mechanisms of radiation resistance, Zhou et al. [17] performed a genome-scale CRISPR Cas9 knockout screen in an NPC cell line under a fractionated irradiation regimen. They identified 210 genes considered radiosensitizer genes, whose loss increases radiosensitivity, and post-gene ontology analysis revealed FBLN5, FAM3C, MUS81, DNAJC17, and CALD1 were the highly enriched genes. Similarly, radiation resistance genes are those whose loss confers radiation resistance, and these genes include TOMM20, SNX22, PSIP1, TLN1, CDKN2AIP, and SP1. However, the authors did not evaluate the effect of depleting the gene that conferred radiation resistance using preclinical models. Overall, these studies successfully provide a mechanism-anchored path to precision platinum/radiation therapy by providing clinically testable targets and tissue biomarkers to personalize medicine in HNSCC. Despite efforts to complete genome-wide CRISPR screens focused on either dependency or treatment perturbation, few studies have validated their findings using in vivo preclinical models. While verification through in vitro experiments is informative, the lack of in vivo validation prevents us from capturing the mechanistic interactions within the host that reflect the complexity of the tumor microenvironment, including stromal and immune cells and endothelial cells that influence therapeutic responses. Therefore, addressing this limitation will be important in order to advance CRISPR-identified vulnerabilities toward clinically actionable strategies for addressing HNSCC. Overall, genome-wide screening studies using CRISPR dropout approaches under treatment perturbation with cisplatin or radiation have revealed that HNSCC resistance mechanisms rely on pro-survival signaling, NOTCH pathways, and DNA damage response (DDR) pathways that regulate the repair of interstrand cross-links. These mechanisms indicate that co-targeting DDR and NOTCH signaling could enhance chemoradiation efficacy in future translational models.

### 3.3. Genes Related to Pharmacological Inhibitors and Involved in Oncolytic Vaccines from Genome-Wide CRISPR Screens

Goto et al. used similar approaches but focused on sensitizing the mTOR inhibitor (mTORi) in OSCC by combining it with targeting agents to suppress resistance pathways [18]. Their group performed a kinome-wide CRISPR Cas9 dropout (loss-of-function) screen in tongue SCC cells post-treatment of INK128 (mTOR inhibitor) to identify genes whose loss enhances mTOR-inhibitor-killing activity. The CRISPR screen was designed as a dropout (loss-of-function) assay, in which sgRNA depletion over time indicated reduced proliferative fitness rather than direct cytotoxicity. The authors selected the second most regulated pathway that relates to the cell cycle pathway as the synthetic-lethal partner for mTORi and demonstrated the synergistic killing activities between the CDK4/6 inhibitor palbociclib and INK128 in both in vitro and in vivo preclinical models. Mechanistically, their study revealed that inhibition of mTOR led to suppression of eIF4G-CCNE1 mRNA complex formation, inhibiting CCNE1 translation and thereby preventing the adaptive CCNE1 level increase that drives rapid resistance to palbociclib. This result reveals the potential of using CRISPR Cas9 screening to identify rational combination treatments for HNSCC. Overall, growth inhibition was demonstrated in both in vitro and in vivo models, as evidenced by BrdU staining, and no apoptotic assays such as cleaved PARP, caspase-3, or TUNEL were performed. Similarly, Wang et al. [19] performed a kinase-related CRISPR Cas9 library screen to identify vulnerabilities that could sensitize HNSCC cells to EGFR inhibition using erlotinib and gefitinib, which constitute the second generation of EGFR inhibitors, while cetuximab represents the first generation of EGFR inhibitor approved for HNSCC treatment. They identified PIK3C2A as the only gene that depleted both gefitinib and erlotinib levels, and transient knockdown of this gene using an siRNA approach sensitized HNSCC cells to gefitinib treatment in vitro. As PIK3C2A pharmacological inhibitors are still underdeveloped, the authors were unable to perform a downstream study to validate the mechanistic link between PIK3C2A depletion and EGFR pathway inhibition. Moving beyond efforts to optimize mTOR or EGFR-targeted strategies for HNSCC, Qiu et al. [21] employed genome-wide CRISPR Cas9 screening to uncover vulnerabilities that enhance responsiveness to oncolytic virotherapy with oHSV-1. They identified the H3K9 methyltransferase SUV39H2 as a key host factor conferring resistance. They demonstrated that genetic depletion or pharmacologic inhibition with OTS186935 increased expression of viral genes (ICP0/ICP4/ICP8) and enhanced viral yield in vitro. In their in vivo study, they combined oHSV-1 with OTS186935 in an immunocompromised-cell-derived xenograft subcutaneous mouse model and found that this combination therapy enhanced anti-tumor efficacy relative to oHSV-1 or an untreated control. Mechanistically, SUV39H2 inhibition reduced H3K9me3 levels and elevated ICP8 levels, facilitating viral replication. To assess immune effects, the authors knocked down SUV39H2 in a syngeneic HNSCC line and treated tumors with oHSV-1. They demonstrated that SUV39H2 loss enhanced oHSV-1 anti-tumor activity and increased intratumoral CD4 and CD8 T-cell infiltration. As cancer cells depend on metabolism to fuel proliferation and confer therapy resistance, numerous studies have begun focusing on inhibiting glutamine metabolism [25]. Recently, Allevato et al. [20] performed a genome-wide CRISPR screen of HNSCC to define mechanisms of resistance to glutamine inhibition and found that GPX4 inhibition via RSL3 sensitized cells to glutamine antagonists or inhibitors (DON) in vitro and in vivo, highlighting the synthetic lethality between glutaminase inhibition with GPX4 inhibition as a promising therapeutic strategy for HNSCC. Collectively, treatment-perturbation screens using targeted therapies such as mTOR inhibitors, EGFR inhibitors, glutamine inhibitors, and oHSV-1 have demonstrated the potential of synthetic-lethal approaches to treating HNSCC by mechanistically targeting mTOR signaling and cell-cycle regulation (INK128–palbociclib synergy), EGFR–PI3K pathway cross-talk (PIK3C2A-mediated TKI resistance), metabolic dependency on glutamine and ferroptosis regulators (GPX4), and epigenetic modulation of viral susceptibility via SUV39H2. Their success in overcoming resistance to these targeted therapies through the use of pharmacological inhibitors or genetically modified approaches provides a rationale for combination strategies in the HNSCC setting.

## 4. Future Directions

Although multiple CRISPR/Cas9 studies have mapped essential genes and therapy modulators in the context of HNSCC, integration between/across studies remains a challenge. The available datasets were compiled using different sgRNA libraries, scoring algorithms, and perturbation conditions (baseline versus cisplatin, radiation, or targeted inhibitor stress). Therefore, addressing direct gene overlap or dependency clustering was not within the scope of this review. However, dependable patterns have emerged, particularly gene vulnerabilities involving the YAP1/WWTR1 via Hippo pathway, NOTCH1, STK19, and mTOR cell cycle mechanism, which may represent essential survival modules in HNSCC. Future studies integrating meta-analyses to leverage dependency metrics such as CERES and Chronos scores will enable us to resolve these overlaps quantitatively and differentiate between basal and stress-induced dependencies to prioritize analysis of robust therapeutic targets across all HNSCC studies.

Neoadjuvant immunotherapy has been adopted in practices following the FDA’s recent approval of pembrolizumab based on KEYNOTE-689 [26], which showed that neoadjuvant pembrolizumab followed by adjuvant pembrolizumab with standard chemoradiation post-surgery improves patient overall survival outcomes versus standard neoadjuvant therapy followed by adjuvant chemoradiation (https://www.fda.gov/drugs/resources-information-approved-drugs/fda-approves-neoadjuvant-and-adjuvant-pembrolizumab-resectable-locally-advanced-head-and-neck (accessed on 4 December 2025)). Accordingly, prioritizing the discovery of vulnerabilities within chemoradiation regimens using genome-scale CRISPR screens to identify radiosensitizers/chemosensitizers and validate them in both syngeneic and humanized HNSCC models that preserve immune context is warranted. This work can provide biomarker-driven combinations that strengthen the benefits of chemoradiation in the perioperative setting.

Next-generation studies should amalgamate functional screens with population genomes to select actionable dependencies. One useful approach is to combine pooled CRISPR knockout/CRISPRi data with TCGA/GENIE/DepMap features that are related to mutations, transcription, and methylation to prioritize context-specific vulnerabilities. In 2023, Dai et al. [27] employed CERES-based algorithms to analyze DepMap CRISPR loss-of-function data regarding HNSCC, uncovering 68 essential genes associated with overall survival and then refining these to 8 independent prognosticators via LASSO (PSMD2, TXNRD1, SLC7A5, CDK6, AP2M1, EGFR, AURKA, and PLK1). In 2025, Pang et al. [28] identified 1511 proliferation-essential genes (PEGs) from DepMap and refined these PEGs to a 7-gene signature (MRPL33, NAT10, PSMC1, PSMD11, RPN2, TAF7, and ZNF335). Both studies functionally validated the essential gene as a driver of proliferation and migration in vitro. However, they did not translate the findings in an in vivo preclinical setting. Despite these efforts, future investigations should incorporate data from single-cell sequencing instead of bulk RNA sequencing and integrate stratification by tumor subsite, HPV status, and immune infiltration phenotype (hot vs. cold tumors), recognizing that HNSCC represents a highly heterogeneous malignancy. Completion of this stratification will help us to improve therapeutic translation across distinct HNSCC subtypes.

Three years ago, Fu et al. [29] performed an in vivo CRISPR screen of a syngeneic mouse model of HNSCC. This work opened up a pathway towards elucidating a microenvironment consisting of immune, fibroblast, endothelial, and neuron cells, which had been missing in in vitro models, via technology that utilizes barcoded libraries in patient-derived xenograft, syngeneic, or humanized models of HNSCC. In 2025, Vu et al. [30] conducted an in vivo CRISPR screen in a transgenic sinonasal tumor model harboring co-mutations in NF1, RASA1, and TP53. The screen uncovered 167 candidate vulnerabilities, including targets involved in DNA/histone methylation and chromatin-remodeling pathways implicated in sinonasal tumorigenesis. Future directions should investigate mechanisms of treatment resistance, particularly in the context of immunotherapy and chemoradiation, so that these targets can be translated into rational combination strategies.

Over the past few years, studies [31,32,33] have increasingly focused on applying CRISPR screening to patient-derived cancer organoids to investigate gene–drug interactions. This trend stems from the fact that patient-derived organoids (PDOs) represent an affordable, higher-throughput, and physiologically relevant preclinical platform for studying cancer biology and evaluating new therapeutic strategies. However, such CRISPR-based functional genomic screening has not yet been conducted in HNSCC PDOs. Replacing 2D monolayer cell lines with PDOs for pooled CRISPR knockout/CRISPRi drug-perturbation screens is warranted. While in vivo CRISPR screens have been reported, PDOs capture much of the in vivo complexity yet remain scalable and genetically tractable, thereby de-risking combination strategies before transferal to animal studies.

Overall, DepMap covered most of the cell lines derived from head-and-neck cancer regions [22]. However, this project did not cover the cell line derived from NPC. Furthermore, this project did not address the genes conferring therapeutic resistance that are commonly provided to HNSCC patients, such as genes conferring resistance to chemotherapy, radiation therapy, and immunotherapy. The corresponding studies [15,16] also highlighted that chemotherapeutic resistance genes are derived from tongue SCC. As HNSCC is known to be a heterogeneous malignancy, CRISPR screens should be expanded beyond tongue SCC and include other cell lines derived from the larynx, hypopharynx, gingiva, buccal mucosa, floor of the mouth, and salivary glands along with HPV-stratified cohorts to differentiate between tobacco smoking and viral oncogene contexts. A key advantage of genome-wide CRISPR screening is its ability to uncover synthetic-lethal partners [34]. Proof-of-concept studies have already shown that pairing an mTOR inhibitor with a palbociclib or glutamine inhibitor and a GPX4 inhibitor or oHSV-1 with the SUV39H2 inhibitor OTS186935 can enhance therapeutic efficacy. Building on this information, studies should systematically evaluate MAPK/ERK, PI3K/AKT, and JAK/STAT3 since HNSCC is driven by these essential oncogenic pathways [35]. Therefore, CRISPR dropout screens conducted under the influence of targeted inhibitors of these mechanisms, followed by pharmacologic validation, constitute a proper translational step equivalent to successful mTOR-centric work. Of note, radiation therapy remains a cornerstone in HNSCC [36]. Thus, there is a need for CRISPR radiosensitizer screens. While Zhou et al. [17] nominated candidate genes associated with radio-resistance, their group did not perform in vitro or in vivo validation. In future efforts, researchers should prioritize employing pooled screens using clinically relevant fractionation to identify radiosensitizing vulnerabilities in DNA-damage response/repair, replication stress, chromatin remodeling, and the hypoxia/HIF-1 pathway. This work should be conducted using downstream orthogonal genetics, small-molecule tools that have been preclinically validated in vitro and in vivo, and biomarker panels to guide patient selection.

## 5. Conclusions

Genome-wide CRISPR Cas9 screening has transitioned from being a discovery technology to a translational tool for research on cancer, including HNSCC. While it has the potential to identify context-specific dependencies, reveal synthetic-lethal partners in particular therapies, and identify biomarker-associated combinations, it also enables multi-omics integration. Despite these advances, this review has highlighted the critical need to expand screens across anatomical subsites and HPV strata thorough a prospective validation pipeline to accelerate precision combinations and improve the clinical outcomes of HNSCC.

## Figures and Tables

**Table 1:** Summary of sgRNA libraries and experimental approaches in CRISPR Cas9-screening studies of HNSCC. The table presents the screening context (either baseline or treatment perturbation), findings, and limitations, highlighting translational opportunities and current gaps across various studies that involve chemotherapy, radiation, targeted, metabolic, and oncolytic contexts.

Study	Cell Lines	Platform	Treatment	Screen Context	Key Dependency or Resistance Genes Identified	Findings	Limitations
Chai et al. [13]	ORL48, ORL115, ORL136, ORL150, ORL153, ORL156, ORL166, ORL174, ORL188, ORL195, ORL204, ORL207, ORL214, ORL215	Human Improved Genome-wide Knockout CRISPR Library v1 (Addgene #67989), 90,709 sgRNAs targeting 18,010 genes; Coverage: 100×; MOI: 0.3	Puromycin (2 µg/mL) for 3–4 days post-transduction; cells harvested on day 18 for genomic DNA extraction.	Baseline (proliferation essentiality)	YAP1, WWTR1 (TAZ), and TEAD1	- Identified 918 fitness genes - Verified that YAP1 and WWTR1 serve as Hippo-pathway drivers in vitro - Highlighted Hippo signaling as a core dependency in HNSCC	- No in vivo validation - Mechanistic link to downstream effectors not tested
Wang et al. [14]	C666-1, NP69	Brunello human-genome-wide CRISPR knockout library (Addgene #73178), targeting 19,114 genes; Coverage: 500×; MOI: 0.3	Puromycin (3 µg/mL) for selection. Cells cultured for 28 days and then harvested	Baseline (dependency screen)	KAT7/BRD1/MEAF6/BRPF1/KAT8, NF-κB signaling (CHUK/IKBKB/IKBKG), de novo purine synthesis (ADSL/GART/PAICS/ATIC), linear ubiquitination (RNF31-RBCK1-SHARPIN and OTULIN), and p53 control (MDM2);	- A total of 711 essential genes identified - Key pathways: MYST acetyltransferases, NF-κB, purine synthesis, and p53 control - Verified that gene depletion reduced NPC cell growth in vitro	- No in vivo validation
Li et al. [15]	TSCCA, CAL27	Human kinase-focused CRISPR library (custom, 5171 sgRNAs targeting 508 kinases; 10 sgRNAs/gene); MOI: 0.3	Puromycin (1 µg/mL) × 7 days, treated with cisplatin (0.125–1 µM) for 48 h	Treatment-Perturbed (cisplatin, 0.125–1 µM)	STK19	- A total of 17 cisplatin-sensitizing kinases were found - Verified that STK19 depletion or pharmacological inhibition by T-12-037-01 enhanced cisplatin sensitivity in vitro through DNA damage with MGMT depletion - Combination of T-12-037-01 and cisplatin suppressed tumor growth in vivo through the synergistic effect.	- Focused on kinases only
Ludwig et al. [16]	UMSCC-49	Human GeCKO v2 pooled CRISPR knockout library (Addgene #1000000048, library v2A); Coverage: ≥300×; MOI: 0.3	Puromycin for 7 days. Cells expanded and treated with cisplatin 0.125 µM daily for 1 or 2 weeks prior to genomic DNA isolation.	Treatment-Perturbed (cisplatin, 0.125 µM)	NOTCH1, SSPO, NCOR1, MARK2, MYCBP, SSPO, RAC1, CCNE1, ULK1, RAC1, CDK5R1, VLDLR, CCNE1, RBL1, DHFR, E2F6, CDK5R1, VLDLR, and MAP2K7	- A total of 207 genes affecting cisplatin response were identified - Verified that NOTCH1 depletion or pharmacological inhibition by γ -secretase inhibitor DAPT sensitizes cells to cisplatin in vitro.	- Only in vitro validation - Mechanism of NOTCH1-mediated resistance not fully defined
Zhou et al. [17]	C666-1	(GeCKO v2.0 pooled library (Shanghai Gikco Co., LTD), targeting 19,050 genes + 1864 miRNAs; MOI: 3	Puromycin 3 µg/mL; 2 Gy radiation for 7 and 14 days.	Treatment-Perturbed (Radiation 2 Gy × 7–14 days)	FBLN5, FAM3C, MUS81, DNAJC17, CALD1, TOMM20, SNX22, PSIP1, TLN1, CDKN2AIP, and SP1	- Identified 210 genes modulating radiosensitivity. - Loss of FBLN5, FAM3C, MUS81, DNAJC17, and CALD1 as radiosensitivity genes - Loss of TOMM20, SNX22, PSIP1, TLN1, CDKN2AIP, and SP1 as radio-resistance genes	- No follow-up mechanistic validation - Lacked in vivo validation
Goto et al. [18]	CAL27	Human kinome CRISPR pooled library (Brunello, RRID: Addgene_75312); Coverage: 650×; MOI: 0.3.	INK128 (10 nM) treatment until ~20 population doublings. Puromycin selection not specified	Treatment-Perturbed (mTOR inhibitor INK128 10 nM)	CDK4/6 signaling	- Identified synthetic lethality between mTOR and CDK4/6 signaling - Combination of INK128 and palbociclib leads to synergistic killing in vitro/in vivo - Mechanism: blocking eIF4G-CCNE1 complex formation.	N/A
Qiu et al. [21]	SCC15	Human GeCKO v2 CRISPR library (A + B sets, Addgene #1000000049), 122,411 sgRNAs targeting 19,052 genes + 1864 miRNAs; Coverage: 500×; MOI: 0.3	Puromycin (2 µg/mL × 7 days). Cells exposed to oHSV-1 for 48 h (control) and 72 h (treatment) for positive/negative selection	Treatment-Perturbed (oHSV-1 infection)	SUV39H2	- Identified SUV39H2 as a host barrier to oHSV-1 - Genetic loss or OTS186935 boosts viral gene expression/replication and T cell infiltration, thereby enhancing anti-tumor efficacy in vitro and in vivo.	N/A
Allevato et al. [20]	CAL33	Brunello whole-genome CRISPR library (Addgene #73178), 76,441 sgRNAs targeting 19,114 genes + 1000 non-targeting controls; Coverage: 300×; MOI: 0.5	Vehicle or DON (0.25 µM) treatment until ~18 population doublings. Puromycin selection not specified	Treatment-Perturbed (Glutamine antagonist DON)	GPX4	- Defined synthetic lethality between GPX4 and glutamine antagonism - Combination of DON and RSL3 induce ferroptotic cell death in vitro/in vivo.	N/A
Wang et al. [19]	UMSCC-49, UMSCC-108, UMSCC-97	Human GeCKO v1 (Addgene #49535) or v2 (Addgene #52961) library and Human Kinase Lentiviral Pool (Sigma HKCRISPR); Coverage: 300×; MOI: 0.3	Puromycin for 7 days. Treated with Erlotinib (1 µM) or Gefitinib (1 µM)—duration not specified	Treatment-Perturbed (EGFR inhibitor Erlotinib or Gefitinib 1 µM)	*PIK3C2A*	- Identified PIK3C2A as synthetic-lethal partner of EGFR-TKI	- No selective PIK3C2A inhibitor available for pharmacologic validation
